# Correspondence, Intelligence, &c.

**Published:** 1822-12-01

**Authors:** 


### XIII.

### CORRESPONDENCE, INTELLIGENCE, &c.

We have again to call the attention of our foreign, and more especially our American brethren, to the thoughtless custom of transmitting packets to us by post. Two parcels (we imagine of journals) lately were presented to us by the postman, one having *3l. 6s.* and the other four pounds sterling, marked as postage. We were obliged, of course, to refuse them. We request those gentlemen who honor us by the transmission of journals or books, to take opportunities of sending them by *private hands*, and thus prevent the disagreeable necessity we are under of returning them to the post-office.

Some of our friends are alarmed lest we should introduce new changes in our journal. They need not be alarmed. We will make no change. The regular limits of the Review (224 pages) shall *never* be encroached upon, or devoted to any other purpose than that of reviews:—but if we choose to give *Extra-limites* papers at the private expense of ourselves or our contributors, surely the public have no reason to quarrel with us for such conduct. If the *Extra Limites* are not good, the subscriber pays nothing for them, and he is not forced to read them—if they contain valuable matters, we think we are entitled to thanks rather than censures for introducing them free of expense.

The Medical Intelligencer has misunderstood us when we alluded to the coincidence of opinion between Drs. Curry and Armstrong respecting typhus fever. That coincidence only referred to the *etiology*, and not to the name or abstract nature of the disease. Both the distinguished physicians in question have attributed the fevers, usually denominated typhus, of late years, to a terrestro-aerial cause, or, in other words, to malaria, rather than to contagion. This is the coincidence to which we alluded, and we adhere to the statement which we then made.

Mr. Godfry's letter respecting a quack calling himself "Dr. Bloomfield," has been received; but it would be useless to pourtray the tricks of such wretches to our professional brethren, and beyond the faculty a medical journal is very little known.

We have it in contemplation to adopt Mr. Kingsley's suggested improvement in respect to the index, at the end of the volume. We thank him for the hint.

Quidnunc's letter (the postage of which, by the way, ought to have been paid by *him*, for it was not worth 1s. 1½*d.* to *us*) has been received. He informs us, among other things, that most of our reviews are anticipated by our cotemporaries. The remedy he proposes would be infinitely worse than the disease. We neither did nor do pretend to give the *earliest* account of books, or to be the first to pronounce sentence on their authors. Our object and end is to give the *fullest* account of publications—and that cannot be done, in a quarterly journal, unless we are granted time to do so. Be it remembered that our vessel is large, heavy laden, and a slow sailer, making only four instead of twelve voyages in the year. And yet we could shew our friend Quid some sturdy proofs that there are many customers for these stale commodities; for notwithstanding the multiplicity and velocity of medical journals, it happens occasionally even yet, that, "news much older than our ale goes round." Be that as it may, we are sorry we cannot comply with Quidnunc's proposed changes in the constitution of our Journal. Our present maxim is "nolumus leges operis mutari," lest there should, at no distant period, be written on the tombstone of the Journal, an epitaph that might probably apply to some of Dr. Quid's patients—"we were doing well—took *farther advice*—and here we are."

P. S. You advise us, dear Quid, to change the title and envelope of the Journal. Pray set the example by *enveloping* your next epistle in a frank. Nothing is so vulgar as those long drawling figures of the postman on the *cover* of a letter. Besides a man of your *mutable* politics must have many patients in Parliament.

The epistle of Anti-Empiricus came to hand, but in it we clearly discern the Quack. No virgin can look more demure (at a marriage or a sermon) than the courtezan;—and none thunder fourth such loud anethemas against *irregulars*, as those whose conduct is guided solely by chacanery and disingenuity—which are more dangerous and disgusting than open undisguised Charlatanism.

Who can *think* one thing, and another *tell*,
Our soul detests him as the gates of hell.

In reply to O. P. we beg to say that we are in opposition to no man, and much less in hostility with any of our cotemporaries. The day, we can confidently predict, is past, when the editors of medical journals shall disgrace themselves and insult their readers by venting their private feelings in personal abuse of their competitors. Urbanity has how superseded illiberality in the medical periodical press.

### NOVELTY.

Our readers will have perceived that Messrs. Jukes and Bush have *invented* an apparatus for extracting poisons from the stomach. The same apparatus was proposed by Boerhaave, and brought to perfection by Dupuytren and Renault in France. The whole apparatus, exactly as described by Mr. Jukes, may be seen in Orfila's toxiocology, vol. I. Sect. 84. So much for the novelty of the thing.

### *Obituary.*

### Dr. MARCET.

This distinguished physician has paid the debt of Nature rather suddenly. The immediate cause of death was arthritic inflammation transferred to the stomach. On dissection the heart was also found in a diseased state.

At the first meeting of the Medico-Chirurgical Society, after the death of Dr. Marcet, an eloquent eulogium was pronounced by Dr. Cooke on the character, talents, and acquirements of Dr. M. the original founder of the society. Sir Gilbert Blane, Dr. Baillie, Mr. Charles Bell, and some other members followed, and a resolution was unanimously passed that a portrait of Dr. Marcet, by one of the first artists, should be procured and hung up in the society's house, as a tribute to his memory, a mark of their esteem, and a memento of the loss they had sustained by his death. In further respect to their deceased member and founder, they unanimously agreed to wave all other business for the evening, and adjourn till that day week.

DR. THOMAS SEEDS.

We have to deplore the death of this very promising and zealous young physician, who died lately of fever at New York. He was the son of Mr. Seeds of Portsea, and, to our knowledge, united very superior talents with a most ardent zeal for, and love of his profession. As a correspondent, as a friend, and as a physician, we have long entertained for this able and estimable young gentleman the most sincere respect and esteem. Would that we were able to pour the balm of consolation into the wounds of his afflicted parent, who has sustained an irreparable loss in the death of such a son!

### ROYAL COLLEGE OF SURGEONS.

THE COUNCIL.

*President*—Sir Wm. Blizard, Kt. Surgeon to the London Hospital, Devonshire Square.

*Vice Presidents*—Henry Cline, Lincon's-In-Fields; and William Norris, Old Jewry.

Thompson Forster, Surgeon to Guy's Hospital, Southampton Street; John Heaviside, George Street, Hanover Square; Sir David Dundas, Bt. Serjeant Surgeon to the King, Richmond; Sir Everard Home, Bt. Surgeon to Chelsea and St. George's Hospitals, and Serjeant Surgeon to the King, Sackville Street; John Adair Hawkins, Great Marlborough Street; Francis Knight, Saville Row; Sir Ludford Harvey, Kt. Surgeon to St. Bartholomew's Hospital Bedford Place; William Lynn, Surgeon to the Westminster Hospital, Parliament Street; John Abernethey, Surgeon to St. Bartholomew's Hospital, Bedford Row; William Lucas, Surgeon to Guy's Hospital, Threadneedle Street; Sir Astley P. Cooper, Bt. Surgeon to the King, and Surgeon to Guy's Hospital, Spring Gardens; Sir Anthony Carlisle, Kt. Surgeon Extraordinary to the King, and Surgeon to the Westminster Hospital; Thomas Chevalier, Surgeon Extraordinary to the King, South Audley Street; John Gunning, Surgeon to St. George's Hospital, and Surgeon Extraordinary to the King, Lower Grosvenor Street; Honoratus L. Thomas, Leicester Place, Leicester Square; Richard C. Headington, Surgeon to the London Hospital, Broad Street Buildings; Robert Keate, Surgeon to St. George's Hospital, Albemarle Street; John P. Vincent, Surgeon to St. Bartholomew's Hospital, Lincoln's-Inn-Fields.

